# Editorial: Pharmacological approaches towards the resolution of neuroinflammation and neurodegeneration

**DOI:** 10.3389/fphar.2022.1132126

**Published:** 2023-01-09

**Authors:** Huazheng Liang, Monokesh K. Sen, Erika Gyengesi, Gerald W. Münch, Faheem Ullah

**Affiliations:** ^1^ School of Medicine, Translational Research Institute of Brain and Brain-Like Intelligence, Tongji University, Shanghai, China; ^2^ Charles Perkins Centre, School of Medical Sciences, Faculty of Medicine and Health, The University of Sydney, Camperdown, NSW, Australia; ^3^ Pharmacology Unit, School of Medicine, Western Sydney University, Penrith, NSW, Australia; ^4^ Neurosurgery, Robert Wood Johnson Medical School, Rutgers University, Piscataway, NJ, United States

**Keywords:** neuroinflammation, neurodegeneration, gut microbiota, plant-based medicine, therapeutic targets

Neuroinflammation in the central nervous system (CNS), when turns chronic, can lead to axonal and neuronal injury (neurodegeneration) resulting in functional deficits including sensory-motor impairment and cognitive decline (Kempuraj et al., 2016; Zhang et al., 2021; Sen et al., 2022). Recent studies showed a complex link among inflammation, functional deficits, and gut-microbiota composition (Sampson et al., 2016; Chen et al., 2021; Liu et al., 2022; Mou et al., 2022). Current therapeutic options (e.g., donepezil, glatiramer acetate) treating neuroinflammatory and neurodegenerative diseases are mostly symptomatic (e.g., cognitive decline), although these are not only expensive but also have unwanted side effects (e.g., diarrhea) and limited efficacy to modulate the gut-microbial composition and ameliorate inflammation (Torkildsen et al., 2016; Yiannopoulou and Papageorgiou, 2020; Goyal et al., 2021). Therefore, searching for new therapeutics, with limited side effects and affordable prices, is an urgent demand to treat neurodegenerative diseases. Naturally derived plant-based under compounds and complementary and alternative therapeutics are getting a lot of attention in recent years due to their widespread efficacy, limited or no side effects, and easy availability (Oke and Tracey, 2009; Kure et al., 2017; Ullah et al., 2017; Atanasov et al., 2021). This Research Topic thus aimed to collect articles investigating the effect of pharmaceutical products derived from natural products against neuroinflammatory and neurodegenerative conditions.

**FIGURE 1:**
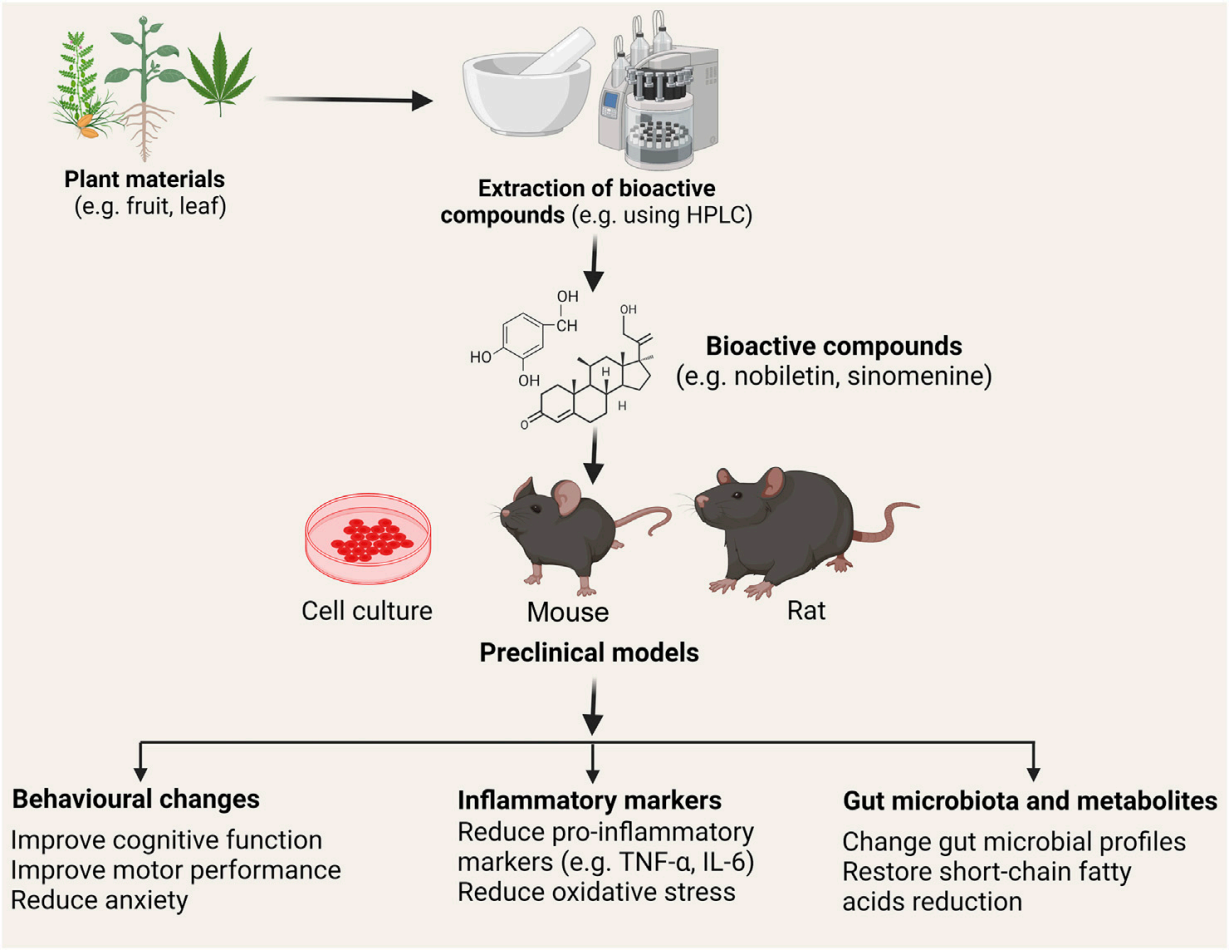
Schematic of the use of plant-derived bioactive chemicals in mitigating inflammation. Various plant (e.g. *Citrus depressa, Sinomenium acutum*) materials including leaves, flower, stems, fruits contain naturally derived anti-inflammatory bioactive compounds. These compounds (e.g., nobiletin, sinomenine, cannabidiol, and tanshinone IIA) are extracted (commonly using homogenization and high-performance liquid chromatography; HPLC analysis) and tested *in vitro* and *in vivo* to investigate the effect in the resolution of neuroinflammation and neurodegeneration. Studies showed that treatment with the bioactive compounds improved behavioural deficits (e.g., motor, anxiety, and cognition), reduced pro-inflammatory markers such as tumour necrosis factor-α (TNF-α) and interleukin-6 (IL-6), and alters gut microbial profiles and their metabolites (e.g. short-chain fatty acids). Following clinical validation and approval, these pharmaceuticals’ compounds can be used in treating neuroinflammatory and neurodegenerative diseases such as Alzheimer’s disease, Parkinson’s disease, and multiple sclerosis. **Note**: Chemical structures of bioactive compounds are given only for illustration purposes and may not match the actual structures of the compounds.

Postoperative cognitive dysfunction (POCD) is a common neurologic complication after anesthesia and surgery, whose incidence varies due to the different natures of surgery. Most patients with POCD will recover in a short time, but some remain symptomatic for a few months or a year. The predominant pathologic mechanism of POCD is peripheral inflammation induced neuroinflammation (Li et al., 2022). The study by Sun et al. showed that nobiletin (NOB) - a flavonoid found in citrus fruits, such as *Citrus depressa*, an enhancer of the retinoic acid receptor-related orphan receptors (RORs) whose activation leads to increased expression of the core clock gene Bmal1, was tested for its effectiveness in alleviating neuroinflammation in a mouse POCD model (Figure 1). It was found that NOB pre-treatment at 1 and 10 mg/kg effectively suppressed the level of a pro-inflammatory marker interleukin-6 (IL-6) in the peripheral blood in a CD1 mouse POCD model 24 h after the surgery. NOB also dose-dependently decreased levels of pro-inflammatory factors like tumour necrosis factor-α (TNF-α) and IL-1β in the prefrontal cortex. In terms of behavioural changes, NOB at 10 mg/kg significantly improved the recognition index in the novel object recognition test and locomotor activity in the open field test. In addition, it mitigated the anxiety state of POCD model mice. At the mRNA level, NOB also reversed the decrease of the clock gene (e.g., Rora, and Rory) expression induced by surgery. In combination with the increased level of brain and muscle aryl hydrocarbon receptor nuclear translocator-like protein 1 (Bmal 1) after NOB pre-treatment and the negative correlation between clock protein Bmal 1 with TNF-α, likely NOB suppressed the expression of pro-inflammatory cytokines through the ROR-Bmal one pathway. However, this is not confirmed in their study. An interesting note is that 30 mg/kg and 50 mg/kg NOB increased the level of IL-6 in the hippocampus although the POCD model had similar levels of IL-1β and TNF-α to control mice. Another research article by Zheng et al. investigated whether diabetes-induced cognitive impairment (DCI) was attenuated by a lipophilic bioactive compound purified from *Salvia miltiorrhiza* Bunge, tanshinone IIA (TAN), by modulating the gut-brain axis. This study found that TAN treatment decreased levels of fasting glucose in the blood, serum inflammatory markers (e.g., TNF-α), and enhanced cognitive functions (assessed by the Morris water maze) of diabetic rats. In addition, a reduction of tight-junction proteins (ZO-1 and occludin) was observed, which altered the integrity of the blood-brain barrier. Gut microbiome sequencing of TAN-treated diabetic rats showed a non-significant negative correlation between serum FBG and the abundance of *Bacteroidetes* like *B. dorei, Lachnoclostridium sp*. YL32, and *Clostridiodes difficile* at the species level. These gut microbiome changes have been associated with the restoration of short-chain fatty acids (e.g., butyric acid)—a key regulator of neuro-endocrine function (Silva et al., 2020) (Figure 1).

In search of naturally derived anti-inflammatory and neurodegenerative products, a comprehensive review by Hong et al. discussed the pharmaceutical potentials of Sinomenine a natural product of *Sinomenium acutum*. This study covered the beneficial and detrimental aspects of Sinomenine. Moreover, this study also covered the mode of action of Sinomenine showed its neuroprotective effect by reducing oxidative stress, neuroinflammation, and neuronal apoptosis. Hong et al. also outlined the role of Sinomenine in several CNS diseases including cerebral ischemia, intracerebral hemorrhage, traumatic brain injury, Alzheimer’s disease (AD), Parkinson’s disease, neuronal hyperactivity, depression, multiple sclerosis, morphine dependence and glioma by taking into consideration of its dosage and possible mechanisms of action in both *in vitro* and *in vivo* models (Figure 1). Another review by Coles et al. summarized the evidence of phytocannabinoids in managing AD. AD is the most common form of dementia and affects millions of people worldwide and searching for new pharmaceutical products is key to reduce sufferings or even to cure the disease (Yiannopoulou and Papageorgiou, 2020). Coles et al. demonstrated that the endocannabinoid system (ECS), which includes endocannabinoids like cannabidiol (CBD) and Δ9-tetrahydrocannabinol (THC), cannabinoid receptors one and two (CB1R and CB2R), as well as enzymes for the biosynthesis or degradation of endocannabinoids, was altered in AD patients. CBD not only inhibits tau hyperphosphorylation in PC12 neuronal cells through reducing glycogen synthase kinase 3*β*, but also increases cell survival by downregulating lipid peroxidation, production of reactive oxygen species and nitric oxide, and A*β* production (Figure 1). The latter is completed through suppressing the activity of *β*-secretase and restoring mitochondria function in AD. As a result, A*β* induced neuroinflammation is alleviated. THC also protects neurons from A*β* induced cytotoxicity by inhibiting A*β* aggregation and weakly suppressing the activity of *β*-secretase. In addition, CBD and THC show synergistic effects on protecting neurons *in vitro*. Cannabis extracts, either rich in CBD or rich in THC, show benefits to AD mice, evidenced by ameliorated memory deficits and improved neuropathology. However, due to the ease for patients to develop an addiction to these cannabinoids and the limited clinical evidence, the therapeutic potential of this type of compounds need to be further investigated.

Taking these studies together, this Research Topic has provided pharmaceutical evidence of naturally derived products. Treatment with plant-based products have been shown to play diverse immune modulatory activities and to alter gut-microbiota profiles resulting in attenuation of inflammation and amelioration of behavioural deficits. These pharmaceutical products can be drug candidates against neuroinflammatory and neurodegenerative diseases and open a new window for clinical trials upon approval.
